# Pestalotioid fungi: A rare agent of onychomycosis among agriculture workers

**DOI:** 10.18502/CMM.6.2.2839

**Published:** 2020-06

**Authors:** Parismita Borgohain, Purnima Barua, Jagadish Mahanta, Lakhi Ram Saikia

**Affiliations:** 1 Department of Life Sciences, Dibrugarh University, Dibrugarh-786004, Assam, India; 2 Department of Microbiology, Jorhat Medical College, Jorhat-785001, Assam, India; 3 Regional Medical Research Center for Northeast, Indian Council of Medical Research, Dibrugarh-786001, Assam, India

**Keywords:** Hair perforation, Nail infection, Non-dermatophytes, *Pestalotiopsis* spp.

## Abstract

**Background and Purpose:**

Pestalotioid fungi are ubiquitous environmental molds that have received considerable attention in recent times not only because of their role as a plant pathogen but also owing to their high frequency of retrieval from human diseases. Regarding this, the present study was conducted to investigate onychomycosis caused by pestalotioid fungi, commonly considered important phytopathogens causing grey blight disease in *Camellia sinensis*.

**Materials and Methods:**

A total of 122 agriculture workers were enrolled from Assam, India. Direct microscopic examination was carried out using 40% KOH to determine the presence of any fungal element. Further processing of the specimens for the isolation of fungi was performed using the standard protocol. In addition, the keratinolytic potential of the isolates was evaluated by means of the in vitro hair perforation test.

**Results:**

Out of 103 culture-positive samples, non-dermatophyte and dermatophyte molds constituted 82.52% (n=85) and 6.79% (n=7) of the samples, followed by yeasts (n=1, 0.9%) and sterile hyphae (n=10, 9.7%). With regard to the isolated non-dermatophyte molds (82.69%), 4 cases belonged to pestalotioid fungi, such as *Neopestalotiopsis piceana* (n=1), *Pestalotiopsis species* (n=1), and *Pseudopestalotiopsis theae* (n=2). The keratinolytic activity of Pestalotiopsis species showed perforation by disrupting the hair cortex; furthermore, macroconidia were found to be present inside the human hair.

**Conclusion:**

A high rate of NDM isolation may be attributed to constant exposure to adverse environmental and occupational hazards. This study highlighted the importance of “pestalotioid fungi” as the rare etiologic agent of onychomycosis. Another remarkable finding was the keratinolytic potential of *Pestalotiopsis* species, which is unique in this study.

## Introduction

Pestalotioid fungi, comprising *NePestalotiopsis*, *Pestalotiopsis*, and *Truncatella*, belong to the order Xylariales. The recent analysis of the 28S rRNA gene of several strains of *Pestalotiopsis* species described two new genera, namely *NePestalotiopsis* and *PseudoPestalotiopsis*, based on morphological and phylogenetic analyses [ 1 , 2 ]. The genus *NePestalotiopsis* was segregated from *Pestalotiopsis* by Maharachchikumbura et al. [ 1 ] using phylogenetic analysis and morphological differences [ 3 ]. All of these species have distinctive morphology. In this regard, the species belonging to genus *Pestalotiopsis* have lightly-pigmented concolorous median cells, while the species belonging to *NePestalotiopsis* have versicolorous median cells and *PseudoPestalotiopsis* with darkly colored concolorous median cells [ 4 ].

Pestalotioid fungi have received considerable attention in recent years because of their role as a plant pathogen [ 5 ]. *Pestalotiopsis* are common in tropical and temperate ecosystems, often isolated as endophytes and may cause plant diseases [ 5 - 8 ]. These fungi are ubiquitous environmental molds that are recovered from the different diseases of human sinuses, fingernails, and bronchi, eyes, and scalp [ 5 , 9 ].

Clinically, onychomycosis is the fungal infection of the fingernail and/or toenail and the surrounding tissues and is considered a major health and aesthetically disrespectful condition [ 10 - 11 ]. Several fungi have been implicated in onychomycosis, including dermatophytes, yeasts, and non-dermatophyte molds (NDMs). The NDM is a heterogeneous group of organisms, which may cause skin infection and onychomycosis [ 12 ]. In the past, these NDMs were regarded as saprophytic or opportunistic fungi [ 13 ]. Many saprophytic fungi, including *Scopulariopsis brevicaulis, Aspergillus, Fusarium, Acremonium*, and *Scytalidium* species, have been reported as the causative agents of onychomycosis [ 14 - 22 ]. However, infections due to the member of pestalotioid group (e.g., *NePestalotiopsis*, *Pestalotiopsis*, and *PseudoPestalotiopsis*) have been rarely documented [ 23 ].

Tea is a major agro-industrial product in Assam, India. The leaves of the tea plant are plucked manually by workers for further processing. Green tea leaf pluckers are most vulnerable to the development of fingernail infections since they use bare fingertips to pluck the moisture-laden small tender shoots. Continuous plucking for years causes repeated injuries to the nail and subsequently exposes the traumatized nails to a variety of phytopathogens present in tea shrubs. The toenails are also affected as the pluckers, in case of remaining bare-footed or using open footwear, are exposed to soil, rainwater, and the trunks of tea plants [ 24 ]. With this background in mind, the present study addressed onychomycosis caused by pestalotioid fungi, which is commonly considered an important phytopathogen causing grey blight disease in tea (*Camellia sinensis*).

## Materials and Methods

**Study population and sample collection**

Agricultural (i.e., rice field workers), tea garden, and horticultural workers with either toenail or fingernail deformities and/or discoloration due to clinical onychomycosis were sampled after taking informed written consent. Over a period of one year (i.e., 2017-2018), a total of 122 cases were enrolled from Assam.

**Mycological examination**

The nail scrapping and/or clipping were collected on a clean and dry black paper after the proper cleaning of the affected site with 70% alcohol using nail clippers or blade and then placed into ziplock bags. The samples were transferred to the laboratory and processed at the earliest time as per English criteria [ 25 ]. All the samples were examined for fungal elements using 40% potassium hydroxide (KOH) and processed for culture in Sabouraud dextrose agar, with and without cycloheximide and chloramphenicol (*HiMedia*). Subsequently, 40% of the KOH mount of the nail samples were kept in a moist chamber for microscopic examination after 24 h. The growth of any dermatophytes, NDMs, and yeasts with positive KOH mount was regarded as the evidence of fungal nail infection. 

The identification of the isolates was performed based on macroscopic and microscopic features. Important observations, such as the duration of growth, colony morphology, and pigment production, were noted for analytical purposes. Lactophenol cotton blue mount and slide cultures were also put up for microscopic identification. The growth of any yeasts was identified by the conventional procedures. The molecular characterization of the selected isolates was carried out in the National Culture Collection of Pathogenic Fungi (NCCPF), Chandigarh, India, by sequencing the internal transcribed spacer (ITS) region of ribosomal DNA. Genomic DNA was extracted by the phenol-chloroform-isoamyl alcohol method [ 26 ]. 

**Figure 1:**
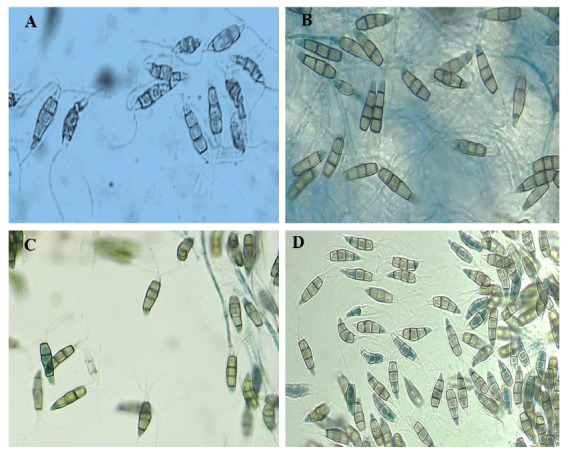
Microscopic morphology showing the macroconidia of pestalotioid fungi; a) *NePestalotiopsis piceana* (MK936319), b) *PseudoPestalotiopsis theae* (MK936320), c) *PseudoPestalotiopsis theae* (MN006270), and d) *Pestalotiopsis* sprcies (MK936321)

The amplification of the complete ITS region was performed using universal primer pair ITS4 (5’-TCCTCCGCTTATTGATATGC-3’) and ITS5 (5’-GGAAGTAAAAGTCGTAACAAGG-3’) [ 27 ]. In addition, polymerase chain reaction (PCR) sequencing was performed for both of the strands using the above-mentioned primers and Big Dye Terminator Cycle ij77 sequencing kit, version 3.1 (Applied Biosystems, Foster City, CA, USA). All the sequencing reaction products were purified and analyzed on an ABI 3130 genetic analyzer (Applied Biosystems). The sequences were then compared with those presented in the GenBank DNA database (https://blast.ncbi.nlm.nih.gov, http://its.mycologylab.org/BioloMICSSequences.aspx, and http://www.westerdijkinstitute.nl/Collections/BioloMICSSequences.aspx) [ 28 ]. All sequences were deposited in the GenBank National Center for Biotechnology Information under accession numbers MK936321, MK936320, MK936319, and MN006270 (Figure 1). For the screening of the keratinolytic potential of the isolates, an in vitro hair perforation test was performed following Salkin et al. [ 29 ].

**Ethical considerations**

Ethical approval was obtained from the Institutional Ethics Committee (human) of Jorhat Medical College, Assam, with a reference number of SMEJ/JMCH/MEU/841/Pt-1/2011/3050. Both verbal and written consent was obtained from the patients.

## Results

A total of 122 clinically suspected cases of onychomycosis were selected from December 2017 to November 2018 from Assam (Table 1). Out of 122 enrolled cases, a total of 140 samples were collected. Most of the participants had toenail involvement (n=94, 77%); furthermore, fingernail involvement and both fingernail and toenail involvement were observed in 19 (16%) and 9 (7%) cases, respectively. Distal and lateral subungual onychomycosis (DLSO) was the most prevalent clinical pattern seen in both fingernail (31%, n=43) and toenail (52%, n=73), followed by total dystrophic onychomycosis (TDO) (fingernail=4%, n=6 and toenail=13%, n=18).

**Table 1:** Demographic profile of the participants

Characteristic	Case	(n=122)
	No.	%
Gender		
Male	65	53.2
Female	57	47
Age group range (years)		
≤20	4	3.2
21-40	69	56.5
41-60	48	39.3
>60	1	0.8
Types of occupation		
Tea garden worker	79	64.7
Rice field worker	10	8.1
Horticulture worker	5	4.09
Tea garden worker+rice field worker	16	13.1
Tea garden worker+horticulture worker	9	7.3
Rice field worker+horticulture worker	3	2.45

It was observed that 97 (69.28%), 6 (4.28%), 14 (10%), and 23 (17.14%) samples were KOH/culture positive, KOH negative/culture positive, KOH positive/culture negative, and KOH/culture negative, respectively. With regard to the culture-positive samples (n=103), NDMs and dermatophyte constituted 82.52% (n=85) and 6.79% (n=7) of the samples, respectively, followed by yeasts (n=1, 0.9%) and sterile hyphae (n=10, 9.7%). Details on the clinical sites of infection and causative fungal agents are outlined in Table 2. The patients had the age range of 17-62 years, and the majority of the samples belonged to the age group of 30-50 years. 

**Table 2:** Frequency distribution of clinical onychomycosis based on the sites of infection and causative fungal agents

Fungal agents	Gender and clinical site				Total
	Fingernail (n=35)		Toenail (n=105)		n (%)
	Male n (%)	Female n (%)	Male n (%)	Female n (%)	
Dermatophyte	2 (15.3)	-	2 (6)	3 (8)	7 (6.8)
Non-dermatophyte	9 (69.2)	20 (91)	25 (81)	31 (84)	85 (82.5)
Yeast	-	-	-	1 (2.7)	1 (.9)
Sterile hyphae	2 (15.3)	2 (9.09)	4 (13)	2 (5.4)	10 (9.7)
Total	13 (100)	22 (100)	31 (100)	37 (100)	103 (100)

**Figure 2:**
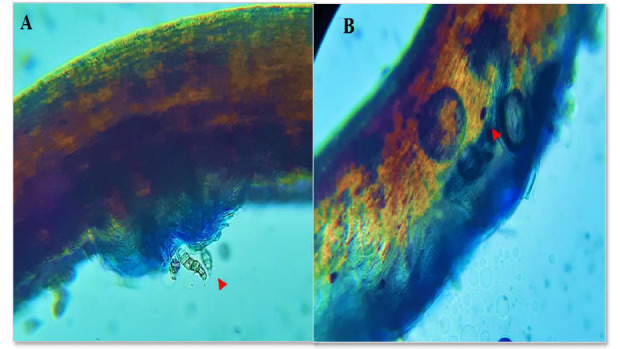
*Pestalotiopsis* species showing hair perforation; a) disruption of the cortex and b) presence of macroconidia inside the human hair

Out of the isolated NDMs (82.5%), 4 cases belonged to pestalotioid fungi, such as *NePestalotiopsis* piceana (n=1), *P*. species (n=1), and *PseudoPestalotiopsis* theae (n=2), which are commonly regarded as phytopathogens (Figure 1a, b, c, and d). With regard to the keratinolytic activity of pestalotioid fungi, only *Pestalotiopsis* species showed the undulation of the cuticle after 10 days of incubation. On the other hand, *N. piceana* and *Ps. theae* demonstrated no keratinolytic activity after 15-20 days of incubation. The best perforation was noted in *Pestalotiopsis* species on the 17^th^ day of incubation as the fungi disrupted the cortex, and macroconidia were found to be present inside the hair (Figure 2a, b). In order to ensure the accuracy of the test, experiments were carried out in duplicates. 

**Figure 3:**
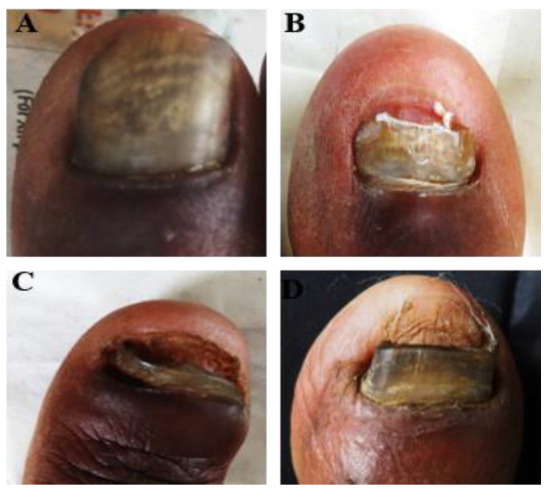
Clinical presentation of nails affected by pestalotioid fungi; a) *NePestalotiopsis* piceana (DLSO), b) *PseudoPestalotiopsis* theae (DLSO), c) *PseudoPestalotiopsis* theae (TDO), and d) *Pestalotiopsis* species (DLSO) DLSO: Distal and lateral subungual onychomycosis, TDO: total dystrophic onychomycosis

The participants with onychomycosis caused by pestalotioid fungi were within the age group of 30-50 years. In this group, two cases were tea garden workers, and the others worked on tea gardens and rice fields. The duration of occupation in the participants ranged from 9 to 22 years with an infection period of 2-9 years. The chief complaints of the subjects were pain and irritation. The most common clinical type was DLSO (n=3), and TDO was detected in only one case (Figure 3).

## Discussion

The results of the present study revealed the higher prevalence of onychomycosis due to NDM in the age group of 30-50 years in females as compared to that in males. The majority of the agricultural workers were female. It can be assumed that besides occupational practice, the domestic activities of females may have increased the burden of trauma to the nails. Similar observations were made by Agarwal et al. [ 30 ] and Barua et al. [ 24 ]. The DLSO was the most prevalent clinical pattern, followed by TDO. This result is in line with those reported by Neupane et al. [ 31 ] and Harika and Usharani [ 32 ].

Recent years have shown the increasing prevalence of onychomycosis due to NDMs across the globe [ 33 ]. In this study, NDMs were the predominant isolates (n=85,82.52%), followed by dermatophytes (n=7,6.79%), yeasts (n=1,0.9%), and sterile hyphae (n=10,9.7%). The isolation rate of NDM may be regarded as high when compared with the rates reported in other studies [ 34 , 35 ]. However, a high rate of NDM (56.6%) was recorded in a study performed by Barua et al. [ 24 ] among the green tea pluckers of Upper Assam. 

With regard to the isolated NDMs (82.69%), 4 species belonged to pestalotioid fungi, such as *N. piceana* (n=1), *Pestalotiopsis* species (n=1), and *Ps. theae* (n=2). These groups of fungi are well-known plant pathogens that rarely cause human infection. In a study conducted in Japan, Monden et al. reported the first case of fungal keratitis caused by *P. clavispora* in humans. In the mentioned study, the patient was treated with topical and intravenous micafungin based on the results of antifungal susceptibility testing [ 36 ].

The identification of pestalotioid fungi is still confusing as morphological and culture characteristics are much more similar among all species. Chen et al. [ 6 ] used morphological techniques and phylogenetic analysis of gene regions (internal transcribed spacer, b-tubulin, and translation elongation factor) to confirm the identification of *PseudoPestalotiopsis* camelliae-sinensis, *N. clavispora*, and *P. camelliae*. The results obtained by Hu et al. [ 37 ] and Chen et al. [ 6 ] showed that the ITS region is less informative than the b-tubulin gene in the differentiation of *Pestalotiopsis* species. The results of the present study were confirmed by sequencing the ITS region with universal primer pairs ITS4 and ITS5 and comparing it with those presented in the NCBI BLAST database (https://blast.ncbi.nlm.nih.gov/Blast.cgi.Literature).

The search in the PubMed revealed no reported case of onychomycosis caused by pestalotioid fungi complex till date. 

In this study, onychomycosis due to pestalotioid fungi complex was identified in 4 agricultural workers within the age group of 30-50 years. Among this group, two cases were tea garden workers, and the others worked on both tea gardens and paddy fields. The most common clinical type was DLSO (n=3), followed by TDO (n=1). The high percentage of toenail infection may be due to poor personal hygiene practices and exposure to water and soil as open sandals are commonly used in this part of the country.

The in vitro hair perforation test revealed the keratinolytic potential of *Pestalotiopsis* species, while other pestalotioid fungi complex, like *N. piceana* and *Ps. theae*, did not show any keratinolytic activity. This type of difference may be due to mutation as in Amphisphaeriaceae family, *Pestalotiopsis* species are rich in asexual morphs with 5-celled conidia [ 1 , 38 ]. However, in most of the species, sexual morph is lacking. According to Katiar and Kushwaha [ 39 ], during perforation, morphological changes, such as conidial germination, the growth of hyphae on hair surface, and in some hair segments, cuticle undulation and cortex disruption, are observed.

Pestalotioid fungi are known to cause grey blight in tea. These fungi have been reported in both temperate and tropical regions, including Japan [ 6 , 8 , 40 - 49 ]. Among these fungi, genus *Pestalotiopsis* has been reported as one of the most devastating phytopathogens in major tea-producing countries [ 40 - 42 , 44 - 46 , 48 , 49 ]. In a study performed in Assam, Rabha et al. [ 43 ] reported that 14-50% of crop loss is due to phytopathogens and *P. theae* as an economically important endophytic fungus causing grey blight in tea plant. In another study carried out in southern India, Joshi et al. [ 50 ] reported that 17% of tea production loss was caused by *Pestalotiopsis* species. In Japan, P. theae and P. longiseta are commonly known to cause grey blight disease in tea. However, infection occurs after plucking or pruning since it enters through the cut parts of the tea plant leaves or stems. 

The continuous plucking, pruning, transplanting, and factory activities of tea garden workers with traumatized nails create an appropriate niche for the phytopathogens present in tea shrubs and soil. It was found that the occupational activites inducing risks in tea gardens, like the use of inorganic fertilizers, pesticides, fungicides, and weedicides, may also contribute to the increase of susceptibility to onychomycosis. 

## Conclusion

The results revealed a high isolation rate of NDM as the potential pathogen of onychomycosis among agricultural workers.. A remarkable finding of the present study was the keratinolytic potential of *Pestalotiopsis* species, which is unique. The organization of awareness campaign, along with the implementation of massive investigation and identification of fungal agents with appropriate antifungal treatment, is the need of hour to reduce the morbid conditions caused by onychomycosis among the agricultural workers.
